# Designing inclusive newborn sequencing research: insights from parents in underrepresented communities

**DOI:** 10.1186/s12920-026-02307-8

**Published:** 2026-01-28

**Authors:** Maya C. del Rosario, Sheyenne A. Walmsley, Barbara W. Harrison, Crystal T. Stephens, Bethany Zettler, Greysha Rivera-Cruz, Priyal Agrawal, Amy Brower, Stephanie Chigbu, Kurt D. Christensen, Casie A. Genetti, Richetta Givens, Nina B. Gold, Inez V. Reeves, Isabella Schichter, Habib Shariat, Sandra Simon, Hadley Stevens Smith, Melissa Uveges, Robert C. Green, Ingrid A. Holm, Stacey Pereira

**Affiliations:** 1 Division of Genetics & Genomics, Department of Pediatrics, Boston Children’s Hospital, Boston, MA USA; 2 Center for Cardiovascular Genetics, Department of Cardiology, Boston Children’s Hospital, Boston, MA USA; 3 Department of Medicine, Mass General Brigham, Boston, MA USA; 4 Ariadne Labs, Boston, MA USA; 5 Department of Pediatrics and Child Health, Howard University College of Medicine, Washington, DC USA; 6 Department of Medicine, The University of Alabama at Birmingham, Birmingham, AL USA; 7 Division of Reproductive Endocrinology and Infertility, Stanford University School of Medicine, Stanford, CA USA; 8 Mindich Child Health and Development Institute, Icahn School of Medicine at Mount Sinai, New York, NY USA; 9 Creighton University School of Medicine, Omaha, NE USA; 10 PRecisiOn Medicine Translational Research (PROMoTeR) Center, Department of Population Medicine, Harvard Pilgrim Health Care Institute, Boston, MA USA; 11 Department of Population Medicine, Harvard Medical School, Boston, MA USA; 12 Manton Center for Orphan Disease Research, Boston Children’s Hospital, Boston, MA USA; 13 Department of Surgery, Mount Sinai Hospital, New York, NY USA; 14 Department of Pediatrics, Harvard Medical School, Boston, MA USA; 15 Department of Pediatrics, Massachusetts General Hospital, Boston, MA USA; 16 Boston College, Connell School of Nursing, Chestnut Hill, MA USA; 17 Department of Medicine, Harvard Medical School, Boston, MA USA; 18 Broad Institute of Harvard and Massachusetts Institute of Technology, Cambridge, MA USA; 19 Center for Medical Ethics and Health Policy, Baylor College of Medicine, Houston, TX USA

**Keywords:** Genome sequencing, Underrepresented populations, Diversity, Ethics, Newborn sequencing

## Abstract

**Background:**

It is essential that studies of genomic sequencing (GS) in newborns and children include individuals from under-represented racial and ethnic groups (URG) to ensure future applications are equitably implemented. We conducted interviews with parents from URG to better understand their perspectives on GS research, develop strategies to reduce barriers to enrollment, and facilitate research participation.

**Methods:**

Semi-structured interviews with 50 parents from URG.

**Results:**

Nearly all parents said they would be interested in participating in an infant GS study. Parents were interested in participating in GS research for reasons including clinical utility, personal utility, and/or family health benefits. Deterrents to enrollment cited by parents were discomfort with enrollment procedures (e.g., not wanting a heel stick), limited emotional bandwidth, unfavorable perceptions of the study, and concerns about potential results. Most parents said they would want to receive all types of genetic results, including actionable and non-actionable, as well as childhood- and adult-onset.

**Conclusion:**

Our findings demonstrate that parents from URG are interested in participating in GS research. Based upon these findings, we provide recommendations for designing GS studies that are responsive to their concerns.

## Background

Previous studies have shown that parents may see value in infant genomic sequencing (GS), but participation in such research has been limited among underrepresented racial and ethnic groups (URG). These disparities may threaten the equitable implementation of genomic research and population screening programs. This paper reports in-depth qualitative insights on the motivations, deterrents, and preferences of URG parents for participation in infant GS research. We offer community-informed strategies to improve enrollment, engagement, and result disclosure approaches for more equitable implementation of genomic screening programs.

## Introduction

When utilized as a screening tool for newborns and children, genomic sequencing (GS) has the potential to identify genetic risks and disorders before symptoms develop, allowing for the early initiation of preventive care and tailored therapy. Research studies that are inclusive of individuals from diverse populations and geographic locations, particularly under-represented racial and ethnic groups (URG) who have been historically excluded from genomics research, are critical to the equitable implementation of GS screening programs. However, historical genetics research practices and ongoing medical racism have created lasting concerns among URG, including mistrust, privacy concerns, and fear of pain from needles involved in sample collection [1–6], resulting in lower rates of enrollment and participation. Some research has identified misconceptions and distrust as barriers to research participation [1, 7], including genetics research. Understanding and addressing the concerns of parents from URG regarding genomic research participation can facilitate engagement of these communities in research and promote future implementation of GS screening programs in a way that benefits infants and families from all backgrounds. These questions are central to the ethical, legal, and social implications (ELSI) of newborn genomic sequencing programs.

From an ELSI perspective, understanding who participates in genomic research, under what conditions, and with what concerns is critical to ensuring that genomic screening programs are socially responsive and equitable. One approach to understanding the perspectives of URGs about a genetic research study is to conduct qualitative interviews of individuals from the communities from which one is engaging prior to recruiting for the study to understand their perspectives about participation in genomic research and develop strategies to overcome participation concerns and barriers. Prior qualitative research to explore willingness to participate in genomics research among underrepresented groups has demonstrated that inquiries about motivators and deterrents to participation did not consistently reflect the investigators’ initial hypotheses [4, 6]. This further underscores the need for exploratory research into factors that influence participation from URGs. This is the approach we took prior to enrolling participants in the second iteration of the BabySeq project. The BabySeq Project is a randomized clinical trial (RCT) of GS in infants [8–11]. In the first iteration of BabySeq we found that exome sequencing of newborns had clinical utility [9, 12, 13] and did not have negative psychosocial effects on families [14]. However, although we offered enrollment to all families in large newborn nurseries and intensive care units that serve diverse populations, the generalizability of our results was limited because the families that chose to enroll were predominantly White, well-educated, and socioeconomically advantaged [15].

To address this lack of diversity, we initiated a second iteration of BabySeq, a new RCT of GS (using genome rather than exome sequencing to increase diagnostic yield and identify copy number variants given its increasing affordability) targeting enrollment to infants from pediatric primary care clinics that serve racially and ethnically diverse communities. Prior to initiating enrollment in the second BabySeq project, to inform the development of our study protocol, recruitment strategies, and disclosure methods, we conducted interviews with parents of children who received care from the BabySeq Project enrollment clinics, as well as a clinic that serves a similar population. Eliciting parents’ views engages key ELSI topics including respect for persons, privacy, and potential psychosocial and family impacts. Here we report interviewees’ hypothetical interest in, motivations for, and deterrents to participating in a study like the BabySeq Project; their preferences for learning about the study and being approached for enrollment; and their attitudes toward return of results and results sharing.

## Materials and methods

### Participants

Participants for this interview study were parents of children who received their primary pediatric care at our future BabySeq Project enrollment sites: Children’s Hospital Primary Care Clinic (CHPCC) at Boston Children’s Hospital (BCH; Boston, MA), Mount Sinai Pediatric Associates Practice of Mount Sinai Hospital (MSH; New York, NY), and Children’s of Alabama Pediatric Primary Care Clinic at the University of Alabama Birmingham’s (UAB) Medical Center (Birmingham, AL). We also interviewed parents of children receiving care at Ambulatory Pediatrics at the Howard University College of Medicine (HUCOM; Washington, DC) due to its long-standing history of providing medical care to individuals from URG and successfully engaging those communities in research. Parents eligible to participate in the interviews were 18 years or older, had at least one child under 18 years of age, and spoke English or Spanish. Due to the project’s focus on enrollment of individuals from URG, we used purposive sampling [16] to offer enrollment to parents of children receiving care at our future BabySeq enrollment sites of self-reported African American or Hispanic/Latino ancestry.

### Recruitment

Primary care providers (PCPs) of eligible parents briefly introduced the study and provided informational flyers in English or Spanish about the study during routine visits. Contact information for interested parents was forwarded to the study team. A member of the study team reached out to interested parents via email and phone to offer participation. We continued recruitment until thematic saturation [16] was reached in the analysis (see below).

### Interviews

A semi-structured interview guide was developed by the study team and reviewed by the BabySeq Community Advisory Board (CAB) for content, language, clarity, and cultural sensitivity. The CAB was convened at the initiation of the second iteration of the BabySeq Project before enrollment began and comprises parents, community leaders, and previous BabySeq participants from the communities from which we are currently enrolling. A brief educational intervention with supporting PowerPoint slides (see Supplemental materials), which included a description of genes, genetic variants, standard newborn screening, and the goals of the BabySeq Project, was delivered prior to interview questions about parents’ perspectives on GS in infants. Demographic information was self-reported at the conclusion of the interview.

Interviews were conducted in English (MCD) or in Spanish by a native Spanish-speaking team member (GRC) by videoconferencing with Zoom, audio-recorded, and the audio files were transcribed following the removal of identifying information. Both interviewers were trained in qualitative research and had no existing relationship with participants. Transcripts of interviews conducted in Spanish were professionally translated into English prior to analysis. Parents were compensated with a $50 gift card for their participation.

### Interview analysis

We used team-based descriptive thematic analysis with a hybrid inductive and deductive approach for codebook and theme development [17, 18]. A codebook of broad deductive codes was developed based on our research questions and the extant literature (e.g., deterrents to enrollment) [1, 4, 7, 15, 19, 20]. Data analysis was aided by the online qualitative analysis software Dedoose (https://www.dedoose.com*)* to manage the coding process. Each interview was coded by three researchers (MCD, SAW, and BWH) using the codebook and then compared for a team-based coding approach. Discrepancies were discussed amongst researchers until consensus was reached for each coded segment. New inductive codes were added to the codebook iteratively, as needed, when predetermined codes did not match our data. We then used an inductive, two-step abstraction process to first identify themes within each broader deductive code, and then categorize the inductively identified themes together into larger, overarching themes [20]. Each segment of text was reviewed by two of four research team members (MCD, SAW, IAH, SP) and all abstractions were discussed until consensus was reached. We conducted interviews until we reached thematic saturation, i.e., when no additional novel themes were identified [16].

## Results

### Study population

We interviewed 50 participants (Table 1) including 30 from HUCOM, 8 from BCH, 7 from MSH, and 5 from UAB. Five interviews were conducted in Spanish, and the rest were in English (90%). Parents primarily self-identified as African American or Black (62%), non-Hispanic (64%), female (92%), were between 20 and 35 years of age (70%), and had some college education (52%). Most parents had more than one child (62%) and most had at least one child who was less than 5 years of age (98%). Most parents had no prior experience with genetic testing (60%). The majority of those who reported average household income indicated that it was below $50,000 (56%).

**Table 1:** Participant characteristics

	Participants, *n* (%)*
Place of Enrollment	
Boston Children’s Hospital	8 (16)
Mount Sinai Hospital	7 (14)
University of Alabama Hospital	5 (10)
Howard University	30 (60)
Gender	
Female	45 (92)
Male	5 (8)
Age (years)	
< 30	23 (46)
30–35	12 (24)
36–40	4 (8)
41–45	10 (20)
46–50	1 (2)
Number of Children	
1	19 (38)
2	11 (22)
3	8 (16)
4	5 (10)
5	2 (4)
6+	4 (8)
Unknown	1(2)
Self-reported Race	
Black, African-American, or African	31 (62)
White	15 (30)
Unknown	4 (8)
Self-reported Ethnicity	
Non-Hispanic	32 (64)
Hispanic	13 (26)
Unknown	4 (8)
Education Level	
Some High School	5 (10)
High School Graduate or GED	19 (38)
Some College	8 (16)
Bachelor’s Degree or Equivalent	13 (26)
Master’s Degree	4 (8)
Unknown	1 (2)
Household Income ($)	
< 15,000–24,999	13 (26)
25,000–49,999	12 (24)
50,000–99,999	5 (10)
> 100,000–150,000	5 (10)
Unknown or declined to respond	14 (28)

*n varies because not all data was collected for all interviews

### Results overview

Nearly all parents said they would be interested in participating in an infant GS study, noting several motivations including clinical utility related to actionability of results, personal utility for psychological preparation, information, or closure, and/or family health benefits. While only a few parents said they would be unlikely to participate in GS research, many parents named reasons that might deter other parents from enrolling, including discomfort with enrollment procedures (e.g., not wanting a heel stick), limited emotional bandwidth, unfavorable perceptions of the study, and concerns about potential results they could receive. See Table 2 for themes of motivations and deterrents to study participation. When asked about results preferences, most parents said they would want to receive all types of genetic results that could be available, including results for preventable conditions, treatable conditions, conditions with childhood onset, conditions with onset only in adulthood, and conditions that are not treatable or preventable.

**Table 2:** Domains, themes, and representative quotes for motivations and deterrents to study participation

Themes	Subthemes	Representative Quotes^a^ (participant ID)
Motivations		
Clinical Utility	Health management	“[Participating in the study] would be a way of learning how to treat him because I [could] know he has a condition and he needs special care for it” (P-19)
	Early treatment/prevention	“I think if there was something that was not normal, that you know, if you know about it early, there could be ways to treat it, or even maybe avoid it.” (P-13)
Personal Utility	Preparedness	“If my baby had this, like the genetic thing that you’re, like, talking about… I could be prepared, like, whether she has it or not, or if she’s a carrier, that her children will have it, so that we would be aware of… so it doesn’t catch us like blindsided.” (P-02)
	Peace of mind	“I want to know. And I don’t want to worry. I want my baby to be healthy.” (P-48)
	Early knowledge	“I would like to know, you know. Anything like, he could be healthy now, and get stuff when he’s older, you know” (P-06)
	Know reason for health condition	“[My friend’s] oldest [child] is autistic… She’s just like, ‘I wish I just knew why, why.’ Because she’s totally fine, and everyone in her family is fine. But then her children have autism… actually… I think it may be helpful” (P-2)
	Benefit of information	“And I think the power of knowing beats the power of anything else” (P-13)
Family Health	Learn family risks	“To know if he’s okay and actually for myself because my partner actually has stuff too… like [if] my parents… passed down stuff too and I don’t know if it’s passed down to me then it’s passed down to my son” (P-43)
	Benefit to future generations	“To see if my baby had a genetic problem and if so if me or my wife could possibly be carriers. I don’t want any of my other children to have genetic problems either” (P-21)
	Lack of family history	“I’m curious to know everything about her, especially since her father isn’t around” (P-31)
	Family health	“If there is something wrong, then we would be able to go ahead and move forward… making sure our child does grow up to be healthy, or our other children, or their children” (P-08)
	Positive family history/explain family health	“[There is a benefit] probably for parents who have lots of health—or particular health issues in their families, they’re probably more likely, maybe, to want to know” (P-09)
Curiosity & Interest	Curiosity	“I’m curious about the health of my child” (P-24)
	Learn about Genetics	“It’ll help me learn a little bit more about, you know, the genetics and stuff” (P-05)
Support Research	Support research	“It would help you guys’ resources.” (P-05)
Community Benefit	Benefit to community	“I believe that it helps with research and I think it is very beneficial for the Black Community, our community, especially because we’re different from white people. There is heart disease, high blood pressure, colon cancer, all of that, there is tons of it, and we need to find out what exactly makes it tick in a sense. Of course it is environmental […] but trying to get it at the root can really help.” (P-32)
Free Testing	Free genetic testing	“I think that it would just be like kind of like ‘of course’ thing. […] we’re offering you a service that is valued at X, Y, and Z. But we’ll be able to take care of your child’s future… that was the biggest thing for me. Like I wouldn’t have to come out of pocket to go pay thousands of dollars for something like this.” (P-03)
Deterrents		
Study Procedures & Emotional bandwidth	Unnecessary testing	“If it’s something that he needs, then yeah, why not? But no, he doesn’t need [genome sequencing]” (P-12)
	Emotional bandwidth	“It’s a lot when you’re a new parent, and you have, you know, vaccines, doctor appointments. Why aren’t they sleeping? Why aren’t they eating? Why are they crying? And if you’re like, ‘Hey, do you want to sign up for this genetic testing?’ And you’re like, ‘I don’t have the capacity to read this information or absorb anything right now’.” (P-13)
	Concern about needles/discomfort	“The only thing I could think about was some parents might be scared of, is maybe they have needles—Like some people really just don’t like to be pricked” (P-03)
Perceptions of the study	Distrust	“[I would want to know] if the study is being used for some malicious activity. Whether it’s for finding out what condition African Americans are more susceptible to and using that information to cause more harm than good. If that is a potential outcome, I would not participate in the study.” (P-03)
	Religious reasons	“We are in the Bible belt. I think there may be sort of religious issues people would have with it. […] I think it would be very clear from the beginning, like, ‘Oh no, that’s not going to work with our belief system’ or whatever.” (P-09)
	Misconceptions	“Many people are focused on what they see on the news or what they listen to in society regarding medical studies and that has a great impact on what they think. So, it’s hard to change their minds because they focus on what social media says and they don’t focus on the benefits.” (P-18)
	Unsure of benefit	“Yes, [the recruitment information] is engaging to participate in the study. But it’s more like—yeah, you’ll get compensated for your time. But I think [the recruitment information] has to be a little bit more of like describing the health benefits of joining this study” (P-14)
Receiving Results	Don’t want to know risk	“Knowing too much information early on can be concerning and alter the way you are with your child and the way you raise your child.” (P-44)
	Discrimination from genetic results	“Would we limit a child’s career choices by doing something like this?” (P-10)
	Wary of false results	“I think there are those that maybe would distrust results, like maybe there could be some false results.” (P-13)

^a^Because parents sometimes talked about more than one theme at the same time, some quotes are relevant to more than one theme

### Receiving information about the study

Based on our preliminary plans for the second iteration of the BabySeq study, we described an infant GS research study. We then asked interviewees how they thought parents should be introduced to such a study. Most parents said the study should be introduced by a clinician that the parent knows, either the child’s PCP or another one of their baby’s clinicians. Other suggested methods for informing parents about the study included by letter, email, or phone call, or via flyers, brochures, or pamphlets at the clinical practice.

Parents were asked how they would like to get more information about the GS study before deciding whether to enroll. Most suggested that the study coordinators provide information using a study website, video, flyers, brochures, or pamphlets. Other parents suggested that study personnel speak directly to the parent’s own PCP or their child’s doctor, connect them with currently enrolled parents, or offer additional opportunities to speak with members of the research staff. Some parents offered suggestions about additional information that would help them decide about participating in the study, such as information about the types of diseases that could be screened and the timeline for their engagement in the study. One parent noted the importance of keeping cultural sensitivity in mind when approaching eligible parents for enrollment, explaining that they felt it was important for study coordinators to understand the cultural background of the potential participants and to approach parents in ways that aligned with their cultural expectations.

### Motivations for enrollment

Most parents said they would be very likely to enroll in an infant GS study like BabySeq. All parents were asked about reasons they might want to or not want to enroll. We identified seven overarching themes of motivations for enrolling (Table 2). One of the most cited reasons was the potential for clinical utility. Some parents suggested early treatment or prevention of health conditions would be the reason they would choose to enroll. One parent specifically noted that “if there was something that was not normal… if you know about it early, there could be ways to treat it, or even maybe avoid it. And I think the power of knowing beats the power of anything else.” (P-13). Others provided less specific reasoning but said they’d be interested in enrolling for purposes of managing their child’s health, such as knowing how to best care for their child.

Another common motivation parents noted was personal utility. Parents’ definition of personal utility including knowing a reason for a health condition, and early knowledge, all of which were described without being linked to specific clinical or health benefits. Other parents echoed their personal utility in the benefit of information: “I think that if they do genetic testing in the beginning, that would prevent a lot of heartaches and a lot of problems that people run into, down in the future” (P-11). Parents also described being able to be prepared, or the potential for GS to provide peace of mind.

Many parents cited family health as a motivation for enrollment. Some parents noted the potential for GS to benefit the health of other members of the child’s family. One parent highlighted this motivation: “If there is something wrong, then we would be able to go ahead and move forward… making sure our child does grow up to be healthy, or our other children, or their children” (P-08). Others noted specific family health benefits, including the possibility for GS information to be beneficial in cases where family health history was unknown, to explain known family history of conditions, or to learn about genetic risk in the family. One parent described how this could be beneficial for reproductive planning and could improve the health of future generations.

### Deterrents to enrollment

A few parents initially indicated that they would personally not be interested in joining an infant GS study. Among these parents, most explained that they felt this testing was unnecessary given all the other routine screening children receive: “If it’s something that he needs, then yeah, why not? But no, he doesn’t need [genome sequencing]” (P-12). Another parent said they would not want to enroll their child because GS utilized as screening in infants is not an established practice. One parent also said they were fearful about this kind of screening but did not explain why, and another expressed that they were unlikely to have enough time to commit to participating in a GS study.

Although the remainder of parents reported they would be likely to join an infant GS study, some offered reasons why they believed that other parents might not enroll. The most cited hypothetical deterrent for a parent not enrolling was not wanting to know the results of testing. Several parents speculated that some parents might be concerned that the results could negatively impact their quality of life, or that a genetic result suggesting a child was at risk for developing a health condition may change the way parents viewed their child.

Distrust and misconceptions about genomic research were mentioned by a few parents as potentially deterring other parents from enrolling. Some parents indicated that other parents may not participate because they wouldn’t fully understand or trust the motives of the research team and would worry that the study could have malicious intent or plans to share data in ways that could harm parents or communities. Others described how the history of mistrust between URG and medical systems could dissuade some from enrolling in a GS study, specifically noting that “the Black community hasn’t had the best relationship [with doctors and healthcare institutions]” (P-39). One parent suggested that misconceptions driven by media portrayal of medical research and negative social media may deter parents from enrolling.

Many parents expressed that logistical issues might deter them, or others, from participating in a GS study. These issues included not having enough available time to devote to the study (e.g., for appointments or surveys), inability to travel for study-related appointments, struggles with taking time off work to participate, and being too busy. Some parents were concerned that collecting a blood sample from their child would cause unnecessary pain or discomfort.

### Return of results

The majority of parents expressed that they would be interested in receiving all types of results described, including results for preventable conditions, treatable conditions, conditions with childhood onset, conditions with onset only in adulthood, and conditions that are not treatable or preventable. When asked why they would like to receive such results, some parents expressed interest in knowing their child’s risk for family planning purposes, and others noted a desire to know their child’s genetic risk to be as prepared as possible to take care of their child as they get older. One parent noted, “I think if there was something that was not normal, that you know, if you know about it early, there could be ways to treat it, or even maybe avoid it.” (P-13).

When asked how they would like to receive their child’s results, most parents said they would prefer to receive results in person, while many said that receiving results via video call would also be acceptable. Fewer parents felt comfortable receiving results via phone or letter. When asked which types of materials they thought could facilitate better understanding, parents were comfortable receiving the written report, while some suggested adding pictures, graphics, or other visual aids, and others suggested a study website or video with additional information. When asked with whom they would share their child’s results, most parents said that they would share results with their child’s doctor and would want the results placed in their child’s medical record for all the clinicians involved in their child’s care to access.

Many parents gave recommendations for the study to provide care after results disclosure, including providing referrals to specialists and follow-up care, and more information about risk-reduction recommendations. Some parents also mentioned that being connected to support and advocacy groups would be personally useful.

### Sharing results with family members

Most parents said they would share their child’s genetic testing results with close family members and would suggest other family members pursue genetic testing, if recommended. Of those parents, some specified that who they would share their child’s results with would depend on both their biological relationship (e.g., first-degree relatives) and emotional closeness with their relative. Parents also mentioned barriers, including family dynamics and location of family members, that may prevent extended family from receiving testing. Almost all parents indicated that if their child had a result that suggested their child was at risk for developing a health condition, it would be important to have themselves and their child’s other parent tested so they could understand their own risk and the risk for their future children. As one parent shared, they would utilize these results “to see if my baby had a genetic problem and if so if me or my wife could possibly be carriers. I don’t want any of my other children to have genetic problems either” (P-21). Only one parent said they would not want to proceed with testing themselves because they felt it would be too late to inform their health management.

## Discussion

In the first iteration of The BabySeq Project [10] we demonstrated that newborn sequencing could identify clinically important health risks [9, 12, 13] in infants without causing psychosocial harm to families [14]. However, like other studies on newborn sequencing to date [21], the first iteration of BabySeq lacked adequate enrollment of individuals from URG. To ensure future applications of GS in infants are equitably implemented, it is critical that we enroll URG participants in GS research. To do so, it is crucial to engage directly with members of URG to not only better understand and address concerns and barriers that may impede their enrollment [4, 22], but also identify factors that may encourage and facilitate participation. In this study, our interviewees were largely positive toward hypothetically participating in a study of infant GS but also identified potential deterrents to enrollment. They expressed preferences regarding various aspects of the study protocol that may not have otherwise been anticipated by our study team and provided suggestions to improve the implementation of GS research and future population-wide genomic screening programs.

These parent interviews directly impacted BabySeq protocol design, implementation, and study logistics. We were able to use many of the suggestions regarding recruitment strategies, including: (1) Involving the child’s PCP in recruitment approach (2), Using outreach by letter, email, phone call, and flyers at the clinical practice (3), Developing a study website and videos (4), Offering multiple opportunities to speak with members of the research staff (5), Creating resources about the types of diseases that could be screened and a timeline for participant engagement in the study, and (6) Hiring study coordinators who understood the cultural background of the potential participants. While we could not connect potential participants with currently enrolled parents, we did film a video of previous participants discussing their BabySeq experience and posted this on the study website. Regarding potential deterrents to enrollment, we modified the sample type from a venous blood draw to a less invasive heel stick but encountered technical limitations to saliva sample collection. We offered remote participation options, and if an extra study visit was required outside of a normal well-baby appointment, we provided compensation for potential costs. For the return of results aspect of the study, we offered video disclosure options and provided the written report as well as a parent-friendly letter to parents and physicians. We also offered family testing through the study if a risk was identified. These decisions were made based on results from this exploratory study as well as regular input from the BabySeq CA B. Many of our findings in this interview study resonate with recurring themes in prior work assessing parents’ attitudes toward infant GS. Our previous research in the first iteration of BabySeq, and others [6], found that parents anticipated clinical and personal utility of newborn sequencing results, which aligns with the motivations for research participation identified in this study [23]. In this study, we found that most parents expressed a preference to receive genomic results related to a wide range of health conditions, including adult-onset only conditions and conditions that are not treatable or preventable, the disclosure of which are currently controversial [19, 24, 25]. These findings are consistent with our previous work [26] and other studies on preferences for genomic results both within and outside the context of infant GS [27, 28]. Likewise, many of the deterrents to enrollment our participants identified reflect actual reasons parents declined the first iteration of the BabySeq Project, such as the additional burden the study could pose, concerns about what results might be received, and potential for discrimination [15]. Interestingly, many of our interviewees did not view these concerns as barriers to their own enrollment in an infant GS study but instead suggested that others may perceive these concerns as barriers. We believe these findings are still important to consider when designing an infant GS study, as these concerns were collected directly from the communities from which we seek to enroll.

Other potential deterrents identified by our interviewees were more closely aligned with established research about reasons for declining research among members of URG, including distrust of and potential for misconceptions about the study [1, 7]. One strategy to foster trust that was emphasized by both interviewees and our CAB was to involve a clinician known to the family to introduce the study. This suggests the inclusion of clinicians who already have an established rapport with families considering participation can help address concerns. Other recommendations for designing infant GS studies that reduce barriers and facilitate enrollment among URG from our research findings cover topics such as sample collection methods, clinical follow-up, support to ease burden on participants, and efforts to reduce distrust and misconceptions; see Table 3.

**Table 3:** Recommendations to facilitate NBSeq research participation among URG

Involve a trusted clinician known to the family.
Offer support for transportation, childcare, and financial well-being.
Use less invasive sample collection methods (i.e., methods that do not involve needles, such as buccal swabs).
Develop a clear pathway for families to access specialized care and support services following the receipt of sequencing results.
Identify strategies to address possible distrust and misconceptions about research, including concerns about potential exploitation or harm.

Our results should be interpreted within the study’s limitations. This work was qualitative, and as such, the goal was not to generate generalizable data, but rather findings that can be transferable to similar contexts, such as other studies enrolling members of URG in newborn and childhood GS or similar research [29]. Another limitation of our study is that the results we have reported are in response to hypothetical questions about interviewees’ interest in participating in an infant GS study; it is possible that they may have felt differently if approached to actually enroll in the study. Similarly, there is the possibility that parents may have been inclined to answer more favorably toward infant GS research in order to appease the interviewer (i.e., social desirability bias). Finally, because our interviewees were parents who agreed to participate in this interview study, it is possible our data reflect the attitudes of individuals who are more open to research participation in general, particularly regarding genomics. Indeed, nearly all our interviewees said they would be interested in participating in an infant GS study while also noting deterrents to participation that may cause other parents to choose not to enroll. Our previous work has demonstrated that actual enrollment in a study may be less than rates suggested when asking potential research participants about enrollment in a hypothetical study [15, 30].

This study aimed to not only include ethical, legal, and social implications (ELSI) of genomics considerations to inform this specific sequencing study, but to also demonstrate integration of ELSI inquiry into the design and implementation of genomics projects. Embedded ELSI work [31] such as this can become a co-productive part of genomic research. This shifts ELSI work from critique from the outside to a constitutive feature of study design. It is important to note that this approach requires researchers to be reflexive and adaptive, as core elements of the planned study may need to be changed to meet the needs, preferences, and values of the communities in question. This way of designing research studies can challenge traditional research structures and processes. If done well, however, this work can lead to enhanced translational value of genomic research. By addressing concerns and barriers, researchers are more likely to enroll participants that are representative of the population, which is necessary not only for justice reasons, but also for scientific rigor. This work can also help build trust for future research, especially with communities who have historically held views distrustful of research. Moreover, bringing these communities into the design of research studies helps promote reciprocity and mutual learning between researchers and participants. Over time, this approach can also help inform institutional practices and policies that prioritize community input and coproduction of study design, leading to more trustworthy and socially responsive genomics research.

Specifically in this study, we sought to integrate the perspectives of people from communities that have been historically underrepresented in genomics research. This field has previously failed to enroll participants representative of the US (or world) population. Many other studies have enrolled populations that have skewed white, wealthy, and well-educated. As such, our study’s goal was to primarily interview parents from traditionally URGs in biomedical research to help drive equity-focused analysis that we hope will improve generalizability of findings [15, 32]. Additionally, integrating the CAB for study design and analysis as well as collaborating with a Historically Black College/University (HBCU) as part of the interviewing process offered a lens that may help facilitate more representative enrollment into research and continue to build trust in research with URGs. Including participant perspectives from URGs connected with Boston Children’s Hospital, Mount Sinai and University of Alabama allowed us to capture insight from geographic regions with varied socioeconomic vulnerabilities. Incorporating feedback and perspectives from racially, economically, and socially URGs allows both our and future projects relating to newborn sequencing to minimize potential harms to all participants and maximize potential benefits as perceived by both researchers and the target populations. Although prior research has been done to assess URG’s perspectives on genomic sequencing, little is known regarding parents’ perspectives on motivators and deterrents to participating in newborn genomic sequencing for apparently healthy infants [13]. By designing a pilot interview study to collect and integrate feedback on a large-scale newborn sequencing project, we aimed to elevate the voices or URGs and advance healthy equity in genomics research.

Our findings support that individuals in URG are interested in genetics research, and direct engagement with both the general population and URG to understand and address concerns and barriers that may impede the enrollment of diverse populations is critical. Earning trustworthiness with URG is essential to produce meaningful research outcomes that will allow diverse populations to confidently access the benefits of genomics. Our findings and recommendations can be used to help design studies of infant GS that are responsive to the concerns among URG that reduce barriers to enrollment.

## Data Availability

De-identified coded transcript excerpts are available upon request to the corresponding author.
